# Awareness, knowledge, and attitudes of the Belgian general population towards paternal perinatal depression: a descriptive cross-sectional study

**DOI:** 10.3389/fpsyt.2024.1455629

**Published:** 2025-01-07

**Authors:** Ayse Akalin, Florence D’haenens, Sandra Tricas-Sauras, Joeri Vermeulen, Dennis Demedts, Ronald Buyl, Maaike Fobelets

**Affiliations:** ^1^ Department of Healthcare, Design, & Technology, Brussels Expertise Centre for Healthcare Innovation (BruCHI), Erasmus Brussels University of Applied Sciences and Arts, Brussels, Belgium; ^2^ Department of Nursing, Faculty of Health Sciences, Düzce University, Düzce, Türkiye; ^3^ Public Health School, CR5 - Department of Social Approaches to Health (CRISS), Université Libre de Bruxelles, Brussels, Belgium; ^4^ Department of Public Health, Biostatistics and Medical Informatics Research Group, Faculty of Medicine and Pharmacy, Vrije Universiteit Brussel (VUB), Brussels, Belgium; ^5^ Department of Life sciences and Medicine, Faculty of Science, Technology and Medicine, University of Luxembourg, Esch-sur-Alzette, Luxembourg; ^6^ Department of Public Health, Mental Health and Wellbeing Research Group, Faculty of Medicine and Pharmacy, Vrije Universiteit Brussel (VUB), Brussels, Belgium; ^7^ Department of Teacher Education, Vrije Universiteit Brussel (VUB), Brussels, Belgium

**Keywords:** DDads questionnaire, health literacy, men, mental health, perinatal depression

## Abstract

**Background:**

Paternal perinatal depression affects 10% of fathers, implying a significant burden on families and public health. A better insight into the population’s health literacy could guide professionals and policymakers in addressing these men and making better use of existing healthcare options. It is also crucial for caregivers, as they play a vital role in identifying symptoms, encouraging help-seeking, and reducing stigma. This study aimed to explore the awareness, knowledge, and attitudes of the general population in Belgium towards paternal perinatal depression, using the validated DDads (Depression in Dads) questionnaire.

**Methods:**

This descriptive, cross-sectional study was conducted between March and May 2020-2022 using convenience sampling. Participants aged over 18 years, with a good understanding of Dutch and residing in the Brussels-Capital Region or Flemish Brabant were invited to participate. Data were analysed using descriptive statistics, Chi² analysis, and independent sample t-test.

**Results:**

A total of 314 participants, including women (n=165) and men (n=149) completed the questionnaire. Anxiousness was the most frequently cited mental health problem for men during their partners’ pregnancy (82.5%) and in the postnatal period (68.5%). Over one-third (37.3%) viewed paternal depression as a ‘normal’ part of the transition to fatherhood. The recommended treatment for paternal depression was mostly non-pharmacological, with family support ranking first (79.9%) and seeking support from informal networks being the most recommended approach (45.2%). New personality characteristics such as withdrawal, cynicism, raging attacks, and irritability, among others, were seen as common symptoms (64.6%). Although 70% of respondents believed that perinatal depression requires specialized treatment, only 39.2% agreed that all men should be screened for depression during pregnancy.

**Conclusions:**

Recognition of paternal depression symptoms was relatively low but higher among females, participants with higher education, and those aware of paternal depression. Professional support recommendations were limited, especially among young people and those without children. The terms ‘paternal perinatal depression’ and ‘perinatal mental health’ are poorly understood. The findings highlight critical gaps in awareness and attitudes, offering valuable insights. Future research should develop tailored interventions to support men’s mental well-being during the perinatal period. Targeted awareness campaigns and healthcare improvements are crucial for addressing this issue.

## 1 Introduction

About 20% of expectant mothers and 4-16% of expectant fathers experience mental health difficulties, such as depression or anxiety, during the perinatal period. A significant increasing percentage is observed three to six months after birth (1–3). While mental health problems may be latent during pregnancy, they may become more pronounced after birth, but they have their origin in pregnancy (2, 4).

The terms perinatal depression and postpartum depression are often used interchangeably, but perinatal depression usually refers to a form of depression that begins from pregnancy recognition or even up to 12 months postpartum (4). Since 50% of postpartum depression begins during pregnancy, it is referred to as perinatal depression. This means that the majority of cases of postpartum depression are already present during pregnancy, but they may not have been diagnosed or treated at that time. Consequently, these cases persist into the postnatal period (5). Therefore, the term “perinatal paternal depressive disorder” refers to a depressive episode of the (future) father within the perinatal period (4, 5).

Paternal perinatal depression refers to depressive symptoms experienced by men during the prenatal or postpartum period (6), although it has not yet been recognized as an official psychiatric disorder (5). It is not fully comparable to maternal perinatal depression and maternal postpartum depression. Depressive symptoms present differently in men and women but depression manifests itself in both cases as a dysphoric mood with reduced activity. While women often experience symptoms such as depressed mood, feelings of hopelessness or pessimism, oversleeping, changes in appetite or weight, the following characteristics tend to predominate in men: withdrawal or avoidance, somatic symptoms, affective rigidity, anger attacks, indecisiveness, cynicism, self-criticism, irritability, partner violence, increased marital conflict, excessive exercise, substance abuse, higher levels of alcohol use, and negative parenting behaviors (7, 8). Because the depressive symptoms of paternal perinatal depression manifest themselves differently, they can disappear in the clinical background. They often occur with anxiety and obsessive disorders, with a series of somatic symptoms and complaints, whether or not together with alcohol and drug abuse (8, 9).

Paternal perinatal depression can occur in resident and non-resident males, as long as they are the biological parent of the child (9). Although higher rates of maternal perinatal depression than those of paternal perinatal depression are observed, there is a positive association between both maternal and paternal perinatal depression (10). The nature and direction of the relationship between paternal perinatal depression and maternal perinatal depression is not yet fully understood, but a moderate positive correlation is observed between maternal perinatal depression and paternal perinatal depression, with 24% to 40% of male partners of women with maternal perinatal depression developing a paternal perinatal depression (1, 11).

It is known that a maternal perinatal depression has serious consequences for both the mother and the new-born. Maternal depression leads to a higher risk of impaired emotional, social, and cognitive development. They are more likely to develop a depression themselves during adolescence or adulthood. There is also an increased risk of developing other child psychiatric disorders such as anxiety disorders (3, 4). However, it is noticeable, that when the child grows up with a father undergoing paternal perinatal depression, there is a greater likelihood of developing externalizing problems, such as behavioral problems and substance abuse (11–13). The effects on the emotional, social, and cognitive development of the child by a parent with maternal perinatal depression or paternal perinatal depression are therefore not entirely parallel. Due to the increased likelihood of developing a psychiatric disorder in the child and its prevalence, it is not unreasonable that maternal perinatal depression is receiving much-needed attention from the community and academia (1, 4). However, because of the negative outcomes on the child as described above, paternal perinatal depression should not be ignored. After all, both forms of perinatal depression cause significant suffering and serious consequences for the child (1–3, 12). Additionally, it is suggested that paternal perinatal depression has a negative impact not only on the development of the child, but also on the well-being, functioning, and relationships of fathers (12, 14), as well as on the entire family system (12, 13). This is relevant as it indicates that paternal perinatal depression extends beyond the negative influences on the development of the child.

Furthermore, men and women have different coping strategies in dealing with these depressive symptoms (7, 8). O’Brien et al. (1) highlight in their review that the most effective treatment methods for fathers experiencing paternal perinatal depression include cognitive behavioral therapy, group interventions, and blended delivery models that incorporate e-support approaches. Similarly, Musser et al. (7) suggest in their literature review that parenting classes, interpersonal therapies, cognitive-behavioral therapy, and pharmacological antidepressant therapy are potential management strategies for paternal postpartum depression. Finally, this review emphasizes the need to develop more evidence-based interventions. These interventions should aim to reduce the risk of paternal perinatal depression and provide fathers with treatments that are timely, appropriate, and acceptable (1, 7). Due to the different expression of the symptoms and the lack of evidence-based interventions, paternal perinatal depression is not always easy to recognize by the fathers’ network, by public opinion, or by healthcare professionals (6–8). In addition, it is difficult to discern between paternal perinatal depression and stress associated with having a new baby in the family. After all, it is normal for the environment and the public opinion that the (upcoming) birth of a baby entails extra stress. Various people will therefore indicate that this is a normal reaction and that “it will pass” (9, 10). This could result in potential sources of help failing to provide the necessary support due to a lack of awareness, thereby exacerbating the stress experienced (8, 10). Factors such as unemployment, economic stressors, lower education levels and work-family conflict can also increase to the stress associated with new fatherhood (1, 12, 13). Therefore, a better insight into the health literacy of the general population regarding paternal perinatal depression could provide valuable guidance to health professionals and policymakers on effectively addressing these men and optimizing the use of existing healthcare resources for fathers experiencing paternal perinatal depression. It is also crucial for caregivers, who play a key role in identifying early symptoms, encouraging help-seeking behavior, and offering ongoing support. The aim of this paper was to explore the awareness, knowledge, and attitudes of the general population in the Brussels-Capital Region and Flemish Brabant towards paternal perinatal depression using the validated DDads (Depression in Dads) questionnaire (7).

## 2 Methods

### 2.1 Design

This study employed a descriptive cross-sectional design using an online survey. The research was conducted following the guidelines outlined in the Strengthening the Reporting of Observational Studies in Epidemiology (STROBE) for observational cross-sectional studies (see **Supplementary File 1**).

### 2.2 Study setting and sampling

The Brussels-Capital Region had 1.222.637 inhabitants and Flemish Brabant, a province of Flanders, had 1.173.440 inhabitants in 2022 (15). The Brussels-Capital Region (bilingual), consisting of 19 municipalities, has a diverse socio-economic population structure with a wide range of nationalities, varying income levels, and living conditions. While several neighbourhoods may find citizens with very limited income, other areas are populated with wealthier expats. Flemish Brabant (Dutch-speaking) has a more homogenous population in terms of nationality, predominantly Belgian, with 65 municipalities (16). In 2021, Brussels remained the region with the lowest average income, while Flemish Brabant was the province with the highest average income per capita (17). In Brussels-Capital Region, 27.8% of the population was considered at risk of monetary poverty (AROP) in 2020, while in Flemish Brabant this rate was 7.3% (18).

### 2.3 Inclusion and/or exclusion criteria

Our participants were women and men aged over 18 years. The inclusion criteria were living in either the Brussels-Capital Region or Flemish Brabant, a province of Flanders, and having a good understanding of Dutch.

### 2.4 Instrument

The DDads questionnaire is based on various pre-existing tools, including an existing questionnaire developed by Highet et al. (19) that focuses on maternal depression, a literature review by Musser et al. (7) on paternal postpartum depression, and the criteria outlined in the Diagnostic and Statistical Manual of Mental Disorders (DSM-5) (5). This instrument was developed and validated by Vermeulen et al. (8) to evaluate the awareness, knowledge, and attitudes of the general population towards paternal perinatal depression. A three-step model was used for its development, which included identifying the content domain, generating items, and constructing the questionnaire. Validation was conducted using two methods: a Delphi method with 17 content experts and cognitive debriefing with 20 lay experts to assess relevance, clarity, wording, and layout. The final version of the scale had a content validity index (S-CVI/Ave) of 0.93, and the face validity index was 1.00, indicating high content and face validity. The validated DDads questionnaire consists of three parts with a total of six questions: a first part with two open ended questions about awareness, a second part with three questions regarding knowledge, and a final part with one question on attitudes. In the part on attitudes, there is one question consisting of 21 items scored on a scale of 1 to 6. Besides the DDads questionnaire, demographic and mental health-related data were collected (**Supplementary File 2**, which has been validated in Dutch and is provided with a free translation in English).

### 2.5 Data collection

Participants were recruited by convenience sampling, similar to the procedure described in Vermeulen et al. (20). Between March-May 2020, each student midwives in the fourth semester of their bachelor’s training were asked to approach at least ten good understanding Dutch participants (residing in the mentioned regions). The students were guided by a researcher (FDH) during their preparation for this task in the course unit ‘Evidence-based Midwifery: Empowering the Midwifery Profession.’ This preparation aimed to provide practical experience in research management, a key objective of their course. The students were provided with verbal and written information about the study and its aims in a standardized manner. In order to facilitate access, the DDads questionnaire was set up online, using LimeSurvey^©^ (LimeSurvey GmbH, Hamburg/Germany). Access to the DDads e-questionnaire was secure, and the software prevented the same user from completing the questionnaire more than once. We avoided distribution in social media, since our team favoured a direct contact to verbally inform participants of the rationale of this study.

### 2.6 Data analysis

Descriptive statistics (frequency, percentages, mean, and standard deviation) were employed to report participants’ demographic characteristics, awareness of mental health problems in the perinatal period, knowledge about symptoms and treatments of paternal depression, and agreement with each item’s attitude statements towards paternal perinatal depression, concerning each part of the DDads questionnaire.

The Chi-square test was used to examine relationships between the participants’ socio-demographic variables and their knowledge regarding paternal depression (symptoms, recommended choices for support and most suitable treatments). Independent t-tests were used to explore the differences in each item’s score on a 6-point Likert scale (ranging from 1 for ‘strongly disagree’ to 6 for ‘strongly agree’) for the attitude statements towards paternal perinatal depression, according to gender. An alpha level of 0.05 was applied when assessing statistical significance.

Statistical analyses were performed using IBM SPSS 28.0^©^ SPSS Statistics for Windows (IBM Corp, Armonk, New York, USA).

Participants responses to the two open ended questions were categorized independently by two researchers (DD and MF) using DSM-5 (5) criteria for: anxiety, coping, emotional lability, anxiousness, interpersonal relationship, psychosomatic problems, tiredness, euthymic mood, and personal life in Microsoft Excel (21).

### 2.7 Ethical considerations

Ethical approval was obtained from the Medical Ethics Committee of UZ Brussel/Vrije Universiteit Brussel (VUB), Belgium on April 24^th^, 2019 (registration number: B.U.N./143/2019/39907). An amendment for this study was approved by the ethics committee on February 28, 2020. All participants were provided with prior information about the aim of the study, their expected involvement, the voluntary nature of their participation, the assurance of anonymity, and that no remuneration would be provided. Participants were also provided with an online informed consent, including and a brief overview of the study’s aims and details, and it was clearly stated that by completing DDads e-questionnaire, informed consent was granted.

## 3 Results

### 3.1 Characteristics of the sample

Out of 541 questionnaires that were distributed, 314 respondents, accounting for 58.0% of participation, were included in the study (n=152 in 2020, n=78 in 2021, n=84 in 2022). The study excluded 212 participants due to missing data, and an additional 15 participants who did not reside in either the Brussels-Capital Region or Flemish Brabant, a province of Flanders.

Most respondents were between 18 and 25 years old (n=141, 44.9%), female (n=165, 52.5%), had completed tertiary education (n=191, 60.9%), and were in wage employment (n=135, 43.0%). Most of them were living in the Flemish-Brabant region (n=220, 70.1%), were either single or in a LAT relationship (n=169, 53.9%) and had no children (n=175, 55.7%). Additionally, 34.1% of the respondents did not have Dutch as their mother tongue. Only 25 respondents (8.0%) had received mental health training, while 71 (22.6%) had sought professional help for mental health problems. Furthermore, 36.3% of respondents had not previously heard of paternal depression before completing the questionnaire (n=114) (**Table 1**).

**Table 1:** Participants’ characteristics (N=314).

Characteristic	Frequency (n)	Percentage (%)
Age (years)		
18-25 26-35 36-45 ≥ 46	141 63 36 74	44.9 20.1 11.5 23.5
Gender		
Female Male	165 149	52.5 47.5
Education level		
Primary level Secondary level Tertiary education Other (not defined)	7 87 191 29	2.2 27.7 60.9 9.2
Employment status		
No income Employee/independent Support income/replacement income Retired	119 156 26 13	37.9 49.7 8.3 4.1
Place of residence		
Brussels-Capital Region Flemish-Brabant	94 220	29.9 70.1
Relationship status		
Married Cohabiting Single/ LAT relationship	124 21 169	39.5 6.7 53.8
Parity		
Parity 0 Parity 1 Parity 2 Parity ≥ 3	175 26 71 42	55.7 8.3 22.6 13.4
Mother tongue		
Dutch French English Arabic Other	207 73 3 14 17	65.9 23.2 1.0 4.5 5.4
Mental health training		
Yes No	25 289	8.0 92.0
Seeking professional help for mental health problems		
Yes No	71 243	22.6 77.4
Having heard of paternal depression		
Yes No	200 114	63.7 36.3

### 3.2 Awareness of mental health problems in the perinatal period

When asked, “What do you consider to be the main mental health problems men experience with themselves during their partners’ pregnancy?” the most frequent answers for main mental health problems were anxiousness (n=259, 82.5%), emotional lability including depression (n=139, 44.3%), and anxiety (n=120, 38.2%). Similarly, regarding the first year following birth, anxiousness was the most frequently cited mental health problem (n=215, 68.5%), followed by emotional lability (n=128, 40.8%), and anxiety (n=66, 21.0%). In addition, these problems were cited with the highest rates in the first answer (out of 4) (**Table 2**).

**Table 2:** Belgian General population's awareness of mental health problems during the perinatal period (N=314).

Main mental health problems experinced by men during their partners’ pregnancy*	Overall answer			1^st^ answer			2^nd^ answer		3^th^ answer		4^th^ answer	
	n	%		n	%^a^		n	%^a^	n	%^a^	n	%^a^
Anxiousness Emotional lability Anxiety Psychosomatic problems Interpersonal relationship Tiredness Personal life Coping Euthymic mood No answer	259 139 120 72 54 41 37 28 7 -	82.5 44.3 38.2 22.9 17.2 13.1 11.8 8.9 2.2 -		104 50 55 36 22 20 22 5 0 -	40.2 36.0 45.8 50.0 40.7 48.8 59.5 17.9 0.0 -		94 42 39 22 18 13 6 13 3 64	36.3 30.2 32.5 30.6 33.3 31.7 16.2 46.4 42.9 -	42 31 20 10 9 4 8 5 2 183	16.2 22.3 16.7 13.9 16.7 9.8 21.6 17.9 28.6 -	19 16 6 4 5 4 1 5 2 252	7.3 11.5 5.0 5.6 9.3 9.8 2.7 17.9 28.6 -
Main mental health problems experienced by men during their partners’ postnatal period**	Overall answer			1^st^ answer			2^nd^ answer		3^th^ answer		4^th^ answer	
	n		%	n		%^a^	n	%^a^	n	%^a^	n	%^a^
Anxiousness Emotional lability Tiredness Anxiety Psychosomatic problems Interpersonal relationship Personal life Coping Euthymic mood No answer	215 128 106 66 61 57 48 20 6 -		68.5 40.8 33.8 21.0 19.4 18.2 15.3 6.4 1.9 -	100 60 56 24 24 21 20 6 3 -		46.5 46.9 52.8 36.4 39.3 36.8 41.7 30.0 50.0 -	69 36 41 28 17 17 13 6 0 87	32.1 28.1 38.7 42.4 27.9 29.8 27.1 30.0 0.0 -	36 18 8 8 19 14 10 6 2 193	16.7 14.1 7.5 12.1 31.1 24.6 20.8 30.0 33.3 -	10 14 1 6 1 5 5 2 1 269	4.7 10.9 0.9 9.1 1.6 8.8 10.4 10.0 16.7 -

*Up to four responses recorded, **Up to four responses recorded, ^a^Row percentage.

### 3.3 Knowledge about paternal perinatal depression

#### 3.3.1 Knowledge about symptoms of paternal depression

When asked, “How can you recognize paternal depression (symptoms)?” The most frequent responses included three symptoms: new personality characteristics (n=203, 64.6%) (withdrawal/avoidance/isolation from social situations, family, or work, tantrums, indecisiveness, self-criticism, cynicism, irritability); change of partner relationship (n=190, 60.5%) (decreased affection, increase in relationship problems, increased hostility, partner violence, decreased commitment within the partner relationship); and negative parenting behavior (n=156, 49.7%) (reduced positive emotions, reduced affection, reduced sensitivity, increased hostility, intrusive behavior and reduced engagement) (**Table 3**).

**Table 3:** Belgian general population’s knowledge about symptoms of paternal depression in relation to socio-demographic variables (N=314)^a^.

Symptoms of paternal depression		Total (n=314)	18-35 (n=204)	≥ 36 (n=110)	*p*		Primary/ secondary (n=94)	Tertiary (n=191)	Other (n=29)	*p*	Brussels-Capital Region (n=94)	Flemish-Brabant (n=220)	*p*
New personality characteristics*	**Yes** **No**	203 (64.6) 111 (35.4)	137 (67.2) 67 (32.8)	66 (60.0) 44 (40.0)	0.206		52 (55.3) 42 (44.7)	129 (67.5) 62 (32.5)	22 (25.9) 7 (24.1)	0.053	58 (61.7) 36 (38.3)	145 (65.9) 75 (34.1)	0.475
Change of partner relationship*	**Yes** **No**	190 (60.5) 124 (39.5)	119 (58.3) 85 (41.7)	71 (64.5) 39 (35.5)	0.283		51 (54.3) 43 (45.7)	122 (63.9) 69 (36.1)	17 (58.6) 12 (41.4)	0.288	63 (67.0) 31 (33.0)	127 (57.7) 93 (42.3)	0.123
Negative parenting behavior*	**Yes** **No**	156 (49.7) 158 (50.3)	104 (51.0) 100 (49.0)	52 (47.3) 58 (52.7)	0.531		40 (42.6) 54 (57.4)	104 (54.5) 87 (45.5)	12 (41.4) 17 (58.6)	0.108	45 (47.9) 49 (52.1)	111 (50.5) 109 (49.5)	0.675
Physical symptoms*	**Yes** **No**	127 (40.4) 187 (59.6)	80 (39.2) 124 (60.8)	47 (42.7) 63 (57.3)	0.545		30 (31.9) 64 (68.1)	82 (42.9) 109 (57.1)	15 (51.7) 14 (48.3)	0.088	37 (39.4) 57 (60.6)	90 (40.9) 130 (59.1)	0.798
Use of alcohol and/or other drugs	**Yes** **No**	98 (31.2) 216 (68.8)	62 (30.4) 142 (69.6)	36 (32.7) 74 (67.3)	0.670		17 (18.1) 77 (81.9)	67 (35.1) 124 (64.9)	14 (48.3) 15 (51.7)	**0.002**	29 (30.9) 65 (69.1)	69 (31.4) 151 (68.6)	0.928
Characteristics			Parity n (%)				Gender n (%)				Having heard of paternal depression before n (%)		
Symptoms of paternal depression			Yes (n=139)	No (n=175)	*p*		Female (n=165)	Male (n=149)		*p*	Yes (n=200)	No (n=114)	*p*
New personality characteristics*	**Yes** **No**		84 (60.4) 55 (39.6)	119 (68.0) 56 (32.0)	0.163		122 (73.9) 43 (26.1)	81 (54.4) 68 (45.6)		**<0.001**	138 (69.0) 62 (31.0)	65 (57.0) 49 (43.0)	**0.033**
Change of partner relationship*	**Yes** **No**		90 (64.7) 49 (35.3)	100 (57.1) 75 (42.9)	0.171		105 (63.6) 60 (36.4)	85 (57.0) 64 (43.0)		0.233	121 (60.5) 79 (39.5)	69 (60.5) 45 (39.5)	0.996
Negative parenting behavior*	**Yes** **No**		61 (43.9) 78 (56.1)	95 (54.3) 80 (45.7)	0.067		79 (47.9) 86 (52.1)	77 (51.7) 72 (48.3)		0.501	103 (51.5) 97 (48.5)	53 (46.5) 61 (53.5)	0.393
Physical symptoms*	**Yes** **No**		57 (41.0) 82 (59.0)	70 (40.0) 105 (60.0)	0.857		72 (43.6) 93 (56.4)	55 (36.9) 94 (63.1)		0.225	90 (45.0) 110 (55.0)	37 (32.5) 77 (67.5)	**0.029**
Use of alcohol and/or another drug	**Yes** **No**		44 (31.7) 95 (68.3)	54 (30.9) 121 (69.1)	0.880		49 (29.7) 116 (70.3)	49 (32.9) 100 (67.1)		0.543	65 (32.5) 135 (67.5)	33 (28.9) 81 (71.1)	0.514

p=Chi² test; ^a^Multiple responses recorded; *See **Supplementary File 2**, question 3.

Bold values indicate p < 0.05.

#### 3.3.2 Relationship between the knowledge of Belgian general population about symptoms of paternal depression and their socio-demographic variables

There was a statistically significant relationship between the respondents’ educational level, gender, and having heard of paternal depression before and their knowledge of paternal perinatal depression some symptoms. More than one-third (n=67, 35.1%) of participants with tertiary education identified the use of alcohol and/or other drugs as symptoms of paternal depression, while this rate was lower (n=17, 18.1%) for those with primary/secondary education levels (p=0.002). Almost three-quarters (n=122, 73.9%) of females considered new personal characteristics as symptoms of paternal depression, while this rate was lower (n=81, 54.4%) for males (p<0.001). Additionally, most of the participants (n=138, 69.0%) who had heard of paternal depression before considered new personal characteristics as symptoms of paternal depression, while this rate was lower (n=65, 57.0%) for those who had not heard of paternal depression (p=0.033). Similarly, almost half (n=90, 45.0%) of participants who had heard of paternal depression before identified physical symptoms as symptoms of paternal depression, whereas this rate was lower (n=37, 32.5%) for those who had not heard of paternal depression (p=0.029). There was no statistically significant relation between age, place of residence, and parity and knowledge of symptoms of paternal depression (p>0.05) (**Table 3**).

#### 3.3.3 Knowledge about support choices of paternal postnatal depression

When asked, “If you (or your husband) had postnatal depression, who would you recommend they go to first? *T*he most frequent responses included informal network (e.g. introspection, partner, family, friends, support group) (n=138, 45.2%) in first rank, and professional mental support (e.g., mental coach, psychiatrist, psychologist) (n=82, 26.9%) in second rank. Additionally, only 10 (3.3%) respondents stated they would not seek/recommend help (**Table 4**).

**Table 4:** Belgian general population's knowledge of support choices for paternal postnatal depression in relation to socio-demographic variables (N=305)*.

Characteristics	Total n (%)	Age n (%)			Education Level n (%)			Place of residence n (%)		
Recommended choices	(n=305)	18-35 (n=204)	≥ 36 (n=110)	*p*	Primary, secondary (n=94)	Tertiary (n=191)	*p*	Brussels-Capital Region (n=94)	Flemish-Brabant (n=220)	*p*
Informal network** Professional mental support** Professional perinatal support** Would not seek/recommend help	138 (45.2) 82 (26.9) 75 (24.6 10 (3.3)	98 (50.0)^a^ 54 (27.6) 38 (19.4)^a^ 6 (3.1)	40 (36.7)^b^ 28 (25.7) 37 (33.9)^b^ 4 (3.7)	**0.030**	46 (51.1) 24 (26.7) 15 (16.7) 5 (5.6)	78 (41.7) 50 (26.7) 54 (28.9) 5 (2.7)	0.101	46 (50.0) 23 (25.0) 18 (19.6) 5 (5.4)	92 (43.2) 59 (27.7) 57 (26.8) 5 (2.3)	0.256
Characteristics		Parity n (%)			Gender n (%)			Having heard of paternal depression n (%)		
**Recommended choices**		Yes (n=139)	No (n=175)	*p*	Female (n=165)	Male (n=149)	*p*	Yes (n=200)	No (n=114)	*p*
Informal network** Professional mental support** Professional perinatal support** Would not seek/recommend help		50 (36.2)^a^ 37 (26.8) 45 (32.6)^a^ 6 (4.3)	88 (52.7)^b^ 45 (26.9) 30 (18.0)^b^ 4 (2.4)	**0.007**	74 (45.7) 44 (27.2) 40 (24.7) 4 (2.5)	64 (44.8) 38 (26.6) 35 (24.5) 6 (4.2)	0.869	80 (41.5) 59 (30.6) 50 (25.9) 4 (2.1)	58 (51.8) 23 (20.5) 25 (22.3) 6 (5.4)	0.071

p=Chi² test; ^*^Number decreased due to unanswered; **See **Supplementary File 2**, question 3; ^a, b^Inter-group differences.

Bold values indicate p < 0.05.

#### 3.3.4 Relationship between the knowledge of Belgian general population about support choices of paternal postnatal depression and their socio-demographic variables

We found a relationship between the respondents’ age and parity, and their views of recommended choices for support regarding paternal postnatal depression. Half (n=98, 50.0%) of participants aged 18-35 recommended informal networks, while this rate was lower (n=40, 36.7%) for those aged ≥36. On the other hand, 33.9% of participants (n=37) aged ≥36 recommended professional perinatal support, whereas this rate was lower (n=39, 19.4%) for those aged 18-35 (p=0.030). Among participants who have children (n=50), 36.2% recommended informal networks, while this rate was higher (n=88, 52.7%) for those without children. Furthermore, 32.6% of participants (n=45) who have children recommended professional perinatal support, whereas this rate was lower those without children (n=30, 18.0%) (p=0.007). There was no statistically significant relation between education level, place of residence, gender, and having heard of paternal depression before and recommended choices for support regarding paternal postnatal depression (p>0.05) (**Table 4**).

#### 3.3.5 Knowledge about treatment of paternal depression

When asked, “What types of treatment are suitable for men experiencing paternal depression?” by far the most frequent responses were family support (n=251, 79.9%), rest/relaxation/spending time with oneself (n=211, 67.2%), sufficient sleep (n=194, 61.8%), talking with and listening to anybody (n=194, 61.8%), and seeking help from a psychologist (n=191, 60.8%), while responders also viewed antidepressants (n=110, 35.0%) as a suitable treatment (**Table 5**).

**Table 5:** Belgian general population’s knowledge about the most suitable treatments for paternal depression in relation to gender (n=314).

Treatment choices for paternal perinatal depression^a^	Answer	Total (n=314) n (%)	Gender		*p*
			Female (n=165) n (%)	Male (n=149) n (%)	
Family support	Yes No	251 (79.9) 63 (20.1)	136 (82.4) 29 (17.6)	115 (72.2) 34 (22.8)	0.247
Rest/spending time with oneself	Yes No	211 (67.2) 103 (32.8)	112 (67.9) 53 (32.1)	99 (66.4) 50 (33.6)	0.787
Sufficient sleep	Yes No	194 (61.8) 120 (38.2)	96 (58.2) 69 (41.8)	98 (65.8) 51 (34.2)	0.167
Talking and listening, with anybody	Yes No	194 (61.8) 120 (38.2)	108 (65.5) 57 (34.5)	86 (57.7) 63 (42.3)	0.159
Seeking help from a psychologist	Yes No	191 (60.8) 123 (39.2)	105 (63.6) 60 (36.4)	86 (57.7) 63 (42.3)	0.283
Exercise/sports	Yes No	158 (50.3) 156 (49.7)	77 (46.7) 88 (53.3)	81 (54.4) 68 (45.6)	0.173
Balanced diet	Yes No	114 (36.3) 200 (63.7)	56 (33.9) 109 (66.1)	58 (38.9) 91 (61.1)	0.359
Antidepressants	Yes No	110 (35.0) 204 (65.0)	63 (38.2) 102 (61.8)	47 (31.5) 102 (68.5)	0.218
Joining a support group	Yes No	110 (35.0) 204 (65.0)	54 (32.7) 111 (67.3)	56 (37.6) 93 (62.4)	0.368
Childcare support	Yes No	105 (33.4) 209 (66.6)	57 (34.5) 108 (65.5)	48 (32.2) (67.8)	0.662
Support from a midwife/nurse	Yes No	98 (31.2) 216 (68.8)	52 (31.5) 113 (68.5)	46 (30.9) 103 (69.1)	0.902
Seeking help from a GP	Yes No	98 (31.2) 216 (68.8)	49 (29.7) 116 (70.3)	49 (32.9) 100 (61.7)	0.543
Get among people	Yes No	90 (28.7) 224 (71.3)	47 (28.5) 118 (71.5)	43 (28.9) 106 (71.1)	0.942
Household support	Yes No	83 (26.4) 231 (73.6)	35 (21.2) 130 (78.8)	48 (32.2) 101 (67.8)	**0.027**
Seeking help from a coach	Yes No	68 (21.7) 246 (78.3)	34 (20.6) 131 (79.4)	34 (22.8) 115 (77.2)	0.635
Seeking help from a obstetrician	Yes No	12 (3.8) 302 (96.2)	8 (4.8) 157 (95.2)	4 (2.7) 145 (97.3)	0.318

p=Chi² test; ^a^Multiple responses recorded.

Bold values indicate p < 0.05.

#### 3.3.6 Relationship between the knowledge of Belgian general population about treatment of paternal depression and their gender

When examining differences by gender regarding “types of treatment suitable for men experiencing paternal depression” the most frequently reported responses by female and male participants were similar to those of the general population, and there was no statistically significant difference between the groups (p>0.05). These responses include family support (female: 82.4%, male: 72.2%), rest/relaxation/spending time with oneself (female: 67.9%, male: 66.4%), sufficient sleep (female: 58.2%, male: 65.8%), talking and listening with anybody (female: 65.5%, male: 57.7%), and seeking help from a psychologist (female: 63.6%, male: 57.7%), respectively (**Table 5**).

### 3.4 Attitudes towards paternal perinatal depression

**Table 6** displays in detail the participants’ degree of agreement with 21 items representing the attitudes of the respondents on paternal perinatal depression. Overall, there was a high rate of disagreement with negative stereotypes regarding paternal perinatal depression, such as the statement “It is only paternal depression when you want to harm or kill the child (90.7%) (Item-10)”, “It is only paternal depression when you are thinking about suicide (91.4%) (Item-11)”, “Paternal depression is a sign of weakness (88.6%) (Item-19)”, “Only career men get perinatal depression (90.8%) (Item-12)” and “Men choose to get paternal depression (91.1%) (Item-20)”. There was generally a high rate of agreement with positive stereotypes about paternal perinatal depression such as “Paternal depression requires special treatment (72.0%) (item-8)” and “Men whose partners are depressed are also at risk for depression (56.1%) (item-13)”. However, among the participants, between 37.3% and 36.6% agreed that paternal depression is a normal event during his partner’s pregnancy and postnatal period, respectively. In addition, 39.2% agreed that “all men should be checked for depression during pregnancy” and 43.0% agreed that this should also be checked after the baby is born (**Table 6**).

**Table 6:** Belgian general population’s attitudes towards paternal perinatal depression and attitude differences according to gender (N=314).

No	Items^a^	Disagree n (%)^c^	Agree n (%)^c^	Don’t know n (%)	Gender		*p*
					Female (n=165) Mean (SD)	Male (n=149) Mean (SD)	
**1. **	It is normal for men to feel depressed during his partner’s pregnancy	186 (59.2)	117 (37.3)	11 (3.5)	3.18 (1.39)	2.92 (1.45)	0.112
**2. **	Paternal depression is a normal part of becoming a parent	183 (58.3)	115 (36.6)	16 (5.1)	3.04 (1.40)	3.04 (1.24)	0.986
**3. **	Knowing how to look after a baby comes naturally to men	239 (76.1)	73 (23.2)	2 (0.7)	2.51 (1.19)	2.78 (1.29)	0.056
**4. **	Men get perinatal depression because they cannot cope with parenthood	219 (69.8)	86 (27.4)	9 (2.8)	2.48 (1.21)	2.80 (1.41)	**0.037**
**5. **	Men get perinatal depression because they have unrealistic expectations	149 (47.5)	146 (46.4)	19 (6.1)	3.34 (1.31)	3.27 (1.32)	0.639
**6. **	Paternal depression did not exist in previous generations	230 (73.3)	64 (20.4)	20 (6.3)	2.26 (1.32)	2.38 (1.48)	0.470
**7. **	Paternal depression is not serious	256 (81.5)	48 (15.3)	10 (3.2)	2.05 (1.15)	2.22 (1.22)	0.221
**8. **	Paternal depression requires special treatment^b^	64 (20.4)	226 (72.0)	24 (7.6)	4.24 (1.27)	4.38 (1.25)	0.345
**9. **	Paternal depression will go away on its own as the baby gets older	163 (51.9)	114 (36.3)	37 (11.8)	3.02 (1.27)	3.21 (1.35)	0.237
**10. **	It is only paternal depression when you want to harm or kill the child	285 (90.7)	16 (5.1)	15 (4.2)	1.53 (0.93)	1.59 (0.87)	0.554
**11. **	It is only paternal depression when you are thinking about suicide	287 (91.4)	18 (5.7)	9 (2.9)	1.53 (0.93)	1.59 (0.87)	0.198
**12. **	Only career men get perinatal depression	285 (90.8)	17 (5.4)	12 (3.8)	1.58 (0.85)	1.72 (0.96)	0.484
**13. **	Men whose partners are depressed are also at risk for depression^b^	109 (34.7)	176 (56.1)	29 (9.2)	3.70 (1.39)	3.70 (1.41)	0.977
**14. **	All men should be checked for depression during pregnancy^b^	178 (56.6)	123 (39.2)	13 (4.2)	3.18 (1.34)	3.12 (1.34)	0.727
**15. **	All men should be checked for depression after the baby is born^b^	166 (52.9)	135 (43.0)	13 (4.1)	3.29 (1.33)	3.18 (1.35)	0.466
**16. **	Men should only be checked for depression when their partner is depressed	215 (68.4)	92 (29.3)	7 (2.3)	2.70 (1.26)	2.63 (1.24)	0.630
**17. **	Men who take medication for paternal depression are weak-willed	270 (86.1)	38 (12.1)	6 (1.8)	1.75 (1.00)	2.03 (1.29)	**0.018**
**18. **	Men with postnatal depression can’t be good fathers	267 (85.0)	43 (13.7)	4 (1.3)	1.81 (1.08)	2.03 (1.40)	0.126
**19. **	Paternal depression is a sign of weakness	278 (88.6)	34 (10.8)	2 (0.6)	1.68 (0.99)	1.98 (1.22)	**0.019**
**20. **	Men choose to get paternal depression	286 (91.1)	22 (7.1)	6 (1.8)	1.55 (0.82)	1.76 (1.19)	0.067
**21. **	Men with paternal depression just want their partner's attention	258 (82.2)	48 (15.3)	8 (2.5)	2.03 (1.29)	2.09 (1.28)	0.696

p= independent sample t-test, "Don't know" was not included in the analyses; ^a^The possible response alternatives for each item are 1–6; ^b^item indicates a positively worded statement; ^c^Strongly (dis)agree, (dis)agree, and slightly (dis)agree have been merged into(dis)agree.

Bold values indicate p < 0.05.

#### 3.4.1 Differences between the attitudes of Belgian general population towards paternal perinatal depression and their gender

When examining the differences in attitude statements towards paternal depression according to gender, it is noteworthy that the mean degree of agreement of males was higher than that of females for items “Men get perinatal depression because they cannot cope with parenthood (Item-4) (p=0.037)”, “Men who take medication for paternal depression are weak-willed (Item-17) (p=0.018)”, and “Paternal depression is a sign of weakness (Item-19) (p=0.019)”. There were no statistically significant differences between gender and other attitude items regarding paternal perinatal depression (p>0.05) (**Table 6**).

## 4 Discussion

The aim of this research was to explore the awareness, knowledge, and attitudes of the general population in the Brussels-Capital Region and Flemish Brabant towards paternal perinatal depression using the validated DDads questionnaire (8). Our results showed that paternal perinatal depression and perinatal mental health terms seem to be poorly understood by the general population. Furthermore, attitudes towards paternal perinatal depression appeared to be diverse.

### 4.1 Awareness of mental health problems in the perinatal period

Focusing on fathers’ mental health in the perinatal period is important for the men themselves, as well as for their partners and children. Insufficient awareness can negatively affect mental health and contribute to the stigmatization of mental illness, fostering discrimination and social judgment. Furthermore, low awareness can hinder access to and utilization of mental health care services, as individuals may feel discouraged from seeking help due to fear of stigma or may not recognize the need for professional support (7, 11, 12). In our study, several mental health problems were underlined by the respondents. Anxiousness was reported by over three-quarters of the sample, and more than one-third cited anxiety, which is a common comorbidity with depression during the perinatal period and a known risk factor for postnatal depression (1). It is noteworthy that depression did not usually emerge as the main mental health problem in the perinatal period. Instead, anxiousness, emotional lability including depression, and anxiety were most commonly mentioned as the first answer out of the four possible options, indicating their prominent impact on men’s mental well-being during perinatal period. This may be because depression is not well understood as a mental health issue during the perinatal period. Therefore, its manifestations, such as anxiety, may be more commonly reported, reflecting a lack of comprehensive awareness regarding the broader spectrum of depressive symptoms. Consequently, there is a need to increase public awareness regarding men’s mental health during the perinatal period. This could potentially contribute to reducing paternal perinatal depression rates and improving overall perinatal mental health, if fathers receive adequate mental health support (11, 13). Remarkably, more than half of the participants in our study had already heard of paternal depression. This suggests a growing awareness of mental health issues, likely influenced by public campaigns, discussions, and prior exposure to mental health information through targeted awareness program.

### 4.2 Knowledge on paternal perinatal depression

First, most respondents were able to identify new personality characteristics (e.g., feeling sad, withdrawal, irritability), which are among the core clinical diagnostic symptoms of a depressive episode specified in the DSM-5 (5). This observation suggests that respondents had only moderate identification of the warning signs for paternal perinatal depression, as evidenced by the fact that only one-third of them recognized alcohol and/or drug use as a symptom of the disorder. Our findings are consistent with studies showing moderate to high levels of literacy on depression-related symptoms (19, 22–25). Psychological difficulties in fathers may be appear as interpersonal conflict, somatic and physical complaints, drug and alcohol abuse, and avoidance behaviour, which could mask underlying psychological issues such as depression or anxiety, potentially influenced by cultural reasons, gender roles, or social image (1, 11). In this context, it is thought that increasing the knowledge level of men’s social support networks about paternal perinatal depression symptoms so they can identify and detect symptoms of paternal depression. To increase social support networks for paternal perinatal depression, strategies could include raising awareness and providing education to family members, friends, and healthcare providers about the symptoms of paternal perinatal depression. This would help these support networks better identify and respond to the needs of affected fathers. Additionally, providing training programs or resources to those who are in close contact with fathers during the perinatal period could further enhance their ability to offer appropriate support (9, 13). This will be one of utmost factors in facilitating early recognition of paternal perinatal depression and encouraging professional help-seeking.

In this study, we found that a higher education level, being female, and having heard of paternal depression before were related to a relatively higher recognition of paternal depression (symptoms). Our results consistent with existent literature (19, 22, 24, 26). These results highlight the importance of targeted educational interventions to improve the recognition and understanding of symptoms of paternal depression, particularly among individuals with lower level of education, males, and those who are not yet aware of paternal depression. By addressing these gaps in knowledge, healthcare providers and support systems can better identify and assist fathers experiencing perinatal depression, ultimately improving the well-being of both fathers and their families.

Second, concerning the most suitable treatments for paternal perinatal depression, the respondents recognized the importance of professional assistance and medical treatment, although non-pharmacological treatment including family support for paternal perinatal depression were also clearly seen as more suitable by the majority of respondents. These results are significant in demonstrating that non-pharmacological treatments are overwhelmingly preferred over the use of antidepressants. Accessing informal sources of help may be easier as they are often perceived as more approachable and trustworthy compared to professional sources. Healthcare providers can enhance support for paternal perinatal depression by engaging informal resources, such as friends, family and community networks. Increasing the awareness of these networks about the symptoms of paternal perinatal depression can help them recognize and respond effectively. The concept of mental health ecosystems, which includes both formal and informal support, provides a useful framework for care providers to connect patients with necessary resources, improving overall mental health outcomes (7, 22, 23). Additionally, research indicates that the general population holds a negative attitude towards seeking professional help for mental disorders (23, 24, 27).

Third, the respondents mostly indicated a preference for informal networks of support if they had themselves (or their partner) paternal perinatal depression, similar to what was found in the literature (24, 28, 29). Various factors may have contributed to this outcome, including the still close-knit social units in Belgium and the immediate and spontaneous support they provided (30, 31). Our convenience sampling may have also contributed to these results, as participants who were more socially connected were likely to engage in the study. Moreover, negative attitudes may provide a plausible explanation for the increased preference for informal support. Considering these findings, it would be valuable to investigate the potential influences on the specific individual preferences. The current study did not investigate into this aspect, and therefore, future research on the factors that affect the utilization of informal sources of help would be particularly beneficial. Furthermore, families or fathers with a more limited informal network can benefit from peer support groups, increased awareness of available mental health resources through healthcare providers, and engagement with non-governmental organizations to enhance access to these resources (7, 13, 14).

The study also revealed a surprising result: despite the frequent contact between midwives, nurses, obstetricians, gynecologists, and GPs with pregnant women and their partners, a psychiatrist or psychologist was almost equally preferred for professional support. This finding suggests that respondents may feel comfortable discussing mental health concerns with both their regular healthcare provider and a mental health specialist. Therefore, it is crucial to ensure that these frontline healthcare providers are adequately equipped to identify and manage fathers with paternal perinatal depression. This can be achieved through training in early screening techniques, fostering communication skills, raising awareness of paternal mental health, and providing regular workshops and peer support groups (8, 23, 29). Additionally, our study indicates that participants who were already aware of paternal depression tended to report a higher frequency of all treatment options for paternal perinatal depression. Similarly, respondents who had previously heard about paternal depression gave each of the support choices for postnatal depression a higher rating. These findings are noteworthy because they demonstrate the importance of mental health knowledge about depression in the general population.

This study found that younger participants were more likely to recommend informal networks as a support option for paternal postnatal depression, while older participants were more likely to suggest professional perinatal support. The findings suggest that younger individuals may be more open to informal support, while older individuals see professional help as a more viable choice for fathers experiencing postnatal depression compared to younger participants. Moreover, in agreement with literature (22, 26), having children also influenced the participants’ knowledge towards paternal postnatal depression. In our study, participants who had children were more likely to recommend professional perinatal support as a form of support for paternal postnatal depression. This may be because individuals with children have firsthand experience with the challenges of parenting and recognize the value of professional support. On the other hand, a lower rate of participants without children recommended informal networks. These individuals may have limited experience with parenthood and might not fully grasp the potential benefits of social support from family and friends during this period.

### 4.2 Attitudes towards paternal perinatal depression

Our results indicate that participants’ attitude towards paternal perinatal depression varied but were generally positive, which is in line with international literature (19, 22, 25, 32). Despite this finding, around two-fifths of the respondents considered paternal perinatal depression in men to be a normal part of pregnancy and the postnatal period, indicating a limited understanding of the issue. The normalization of depressive symptoms during the perinatal period by both men and their support networks can be a barrier for seeking professional help to address these symptoms. Additionally, social expectations of fathers to be self-reliant, stigma surrounding male mental health, and a lack of resources targeted toward fathers exacerbate this issue (14, 19, 22, 23). Therefore, there is a need to demystify this belief. Studies have shown that social support networks including partners, family, and friends who know and believe in the presence of perinatal depression might motivate men to seek professional help and treatment (22, 33). One meta-synthesis also revealed that stigma (self-stigma and others’ perceptions) is one of the primary barriers to seek help for perinatal psychological distress (34). Furthermore, it is concerning that nearly a quarter of respondents believed that men have natural skills to care for a baby, which may normalize the challenges faced during the postpartum period and undermine the seriousness of depressive symptoms. This could potentially discourage men from seeking professional help for paternal perinatal depression. It is important to recognize that all men can experience depression during fatherhood, as it is a challenging time for many individuals (35). Neglecting the signs and seriousness of paternal perinatal depression can create obstacles for fathers in obtaining the necessary treatment to address their condition (1, 11). According to the systematic study conducted by Davenport et al. (35), new fathers experience depression but hesitate to disclose their mental health problems due to fear of judgment, leading them to normalize or conceal their symptoms. This highlights the need for education initiatives aimed at dispelling such prevalent misconceptions within the general population.

Another notable finding of our study is the relatively positive attitude towards screening for paternal depression during pregnancy and the postnatal period, as well as when the partner is experiencing depression. Meanwhile, the literature reports that the general population is positive towards screening women’s mental health in the perinatal period (19, 25, 26). This finding is significant, particularly in the context of healthcare systems that may not actively identify cases of paternal perinatal depression through screening. It is utmost importance for a screening program to be accepted by (future) fathers. Therefore, these results could be especially valuable to policymakers who are considering the implementation of paternal depression screening within a comprehensive perinatal health program. Additionally, screening programs can help encourage some fathers to reflect on their symptoms and recognize them as signs of depression. Although screening helps men identify as having paternal depression, this might reinforce stigma and contribute to poor mental health literacy regarding paternal perinatal depression. Furthermore, this may lead fathers to fear wasting healthcare professionals’ time, avoid seeking help, or wait until reaching a crisis point due to concerns about being prescribed medication (35). It is essential to enhance the general population’s understanding of perinatal mental health and increase their awareness of screening. Moreover, programs aimed at screening for perinatal mental health issues in mothers should also inquire about the father’s mental health. If a father reports distress, it should be followed up with appropriate support and interventions. In addition, most respondents in our study believe that men do not choose to get paternal depression and that paternal depression is not a sign of weakness. These responses indicate respondents’ positive attitude about the emotional assistance men require from their social support networks when confronted to paternal perinatal depression. Nevertheless, there are still some important knowledge gaps and biased attitude towards paternal perinatal depression.

Our study revealed that there were significant differences in some attitude statements based on gender. Specifically, males showed higher agreement than females on items relating to “men’s coping abilities with parenthood”, “the perception of weakness in men who take medication for paternal depression”, and “the belief that paternal depression is a sign of weakness”. These findings suggest the presence of societal expectations and stereotypes regarding men’s emotional experiences during the perinatal period. The belief in traditional gender norms that expect men to be strong might contribute to the perception that men who experience depression during this phase cannot cope with parenthood. Additionally, the stigma surrounding men seeking help for mental health issues may discourage them from addressing their emotional struggles during the perinatal period. Branquinho et al. (22) indicated that negative attitudes about postpartum depression have been more frequently found in males. These results highlight the need for targeted interventions to raise awareness about paternal perinatal depression, challenge harmful stereotypes, and destigmatize help seeking behavior for mental health issues among men. By creating a more supportive environment, such as promoting open discussions about paternal mental health, providing accessible mental health resources, and encouraging community and familial support, men may feel more comfortable seeking assistance (26, 34). This can lead to better mental health outcomes for fathers, and their families.

### 4.3 Strengths and limitations

This is the first study to investigate the awareness, knowledge, and attitudes of the general population towards paternal depression in Belgium, using the validated DDads questionnaire. The results are significant to gain insight into the awareness, knowledge, and attitude towards paternal perinatal depression of the public: they are informal sources of support for fathers and play an important role in encouraging them to seek professional help by including both men and women as participants. However, the study also has several limitations. First, data are collected in Belgium (Brussels and Flanders), and the DDads questionnaire does not include samples from studies conducted in different settings or countries. This may limit the comparison of the results. Secondly, the y data were collected through students who contacted participants within their networks via a link to the online questionnaire. To enhance the trustworthiness of the data, we implemented various strategies including proper preparation for the students as part of the Evidence-Based Midwifery course, secure access to the online questionnaire, and the survey software prevented multiple submissions. Thirdly, participants were recruited from within the students’ own networks, which could create bias, as this approach may only capture a specific demographic (e.g., the middle class, those with computer literacy). These factors limit the generalizability of the findings, and the results should be interpreted with caution given the sociodemographic characteristics of the respondents. The final limitation, we used a convenience sampling method to include participants from Brussels and the Flemish-Brabant population in Belgium; therefore, findings may not be generalizable to the entire population of Belgium.

### 4.4 Implications for practice and future research

The findings of this study hold significant relevance for implications for practice, particularly in Belgium, by shedding light on gaps in awareness, knowledge, and prevailing attitudes toward paternal perinatal depression among the general populace. These insights are instrumental in guiding healthcare professionals and policymakers towards informed decision-making in addressing this issue. Healthcare professionals are urged to heighten their vigilance in recognizing early symptoms of paternal perinatal depression, thereby enabling the prevention of paternal depression and timely implementation of interventions. It is imperative for healthcare systems to broaden their spectrum of services to encompass tailored support mechanisms such as individual, couple, and peer support groups, where fathers can share experiences and receive advice from others who have navigated similar journeys, throughout the pregnancy. Additionally, it is essential to cater to the needs of expectant fathers, as their involvement can positively impact the overall family dynamics.

Furthermore, Ministry of Health and Non-Governmental Organizations should spearhead awareness campaigns targeting the public. These campaigns serve as informal avenues of support for fathers, encouraging them to seek professional assistance for paternal perinatal depression symptoms and highlighting the detrimental effects of untreated mental health issues on family members. In addition to broad public campaigns, targeted information initiatives should be set up through digital media, community outreach programs, and healthcare settings, focusing on specific support networks such as spouses, partners, family members, and parents-in-law, recognizing their potential role in bolstering men’s mental health during the perinatal period. Such comprehensive strategies aim to foster a supportive environment conducive to the mental well-being of expectant fathers and their families. Qualitative research is warranted to explore exactly what influences individuals’ increasing preference for informal support when they themselves (or their partner) have paternal perinatal depression and to determine the factors that influence informal help. Further research in this area is warranted to deepen our understanding and develop evidence-based strategies to support men’s mental well-being during the perinatal period. Research into the role of peer support is also crucial for mitigating paternal perinatal depression. Additionally, understanding how informal networks can better assist fathers is an important area for further investigation. Exploring strategies to strengthen these networks could offer valuable insights into improving mental health outcomes for fathers. Future studies could also explore specifically caregivers’ attitudes to develop a more comprehensive approach. We also recommend conducting studies in other cultural contexts to assess the general population’s awareness, knowledge, and attitudes towards paternal depression using the validated DDads questionnaire. This would facilitate cross-cultural comparisons in healthcare and increase the visibility and discussion of paternal depression in societies. Further studies using different approaches such as mixed methods, longitudinal designs, and qualitative research are essential to investigate this phenomenon.

## 5 Conclusion

Our results mainly highlight the need to increase awareness, which can be addressed through targeted information campaigns and interventions aimed at enhancing awareness and improving attitudes of the Belgian population (in Brussels and Flanders) toward paternal perinatal depression. We found that anxiousness followed by emotional lability, and anxiety were viewed as the main mental health problems experienced by fathers during the perinatal period. Non-pharmacological treatments were more commonly suggested over the use of antidepressant medications. Furthermore, the most preferred choice of support concerning paternal postnatal depression was informal networks. Although some participants considered paternal depression as normal, almost all participants believed that it requires special treatment. Higher educational level, being female, and having heard of paternal depression before were related to a relatively higher recognition of paternal depression. Older age and having children were factors that make individuals more likely to prefer professional perinatal support as a recommended option for paternal postnatal depression. The results also explored the existence of gender-based differences in attitudes towards paternal perinatal depression.

## Data Availability

The raw data supporting the conclusions of this article will be made available by the authors, without undue reservation.
