# Supplementary material for: Awareness, knowledge, and attitudes of the Belgian general population towards paternal perinatal depression: a descriptive cross-sectional study
Source: Front Psychiatry. 2025 Jan 7;15:1455629. doi: 10.3389/fpsyt.2024.1455629 (PMC11753207; doi:10.3389/fpsyt.2024.1455629)
Supplement: Supplementary file 2 [file SupplementaryFile2.docx]

**Supplementary File 2**

**Depression in Dads Questionnaire (DDads)**

**PART 1: AWARENESS**

In this section, we are looking at the awareness of the population about psychological (mental) health issues in men during their partners pregnancy and in the first 12 months after birth.

1. **What would you consider to be the main psychological (mental) health problems men experience with themselves during their partners’ pregnancy? (open question - max. 4 responses)**
2. **In the first year after the baby is born, what do you consider to be the main mental health problems men experience with themselves in this first year? (max. 4 responses)**

**PART 2: KNOWLEDGE OF SYMPTOMS AND TREATMENTS**

In this section, we are looking at the knowledge of the population about paternal depression.

1. **How can you recognise a paternal depression (symptoms)? (multiple answers possible)**

- Change in partner relationship: low affection, increased relationship problems, partner violence.
- Negative upbringing behavior: feeling sad/depressed/miserable (reduced positive emotions), reduced affection, decreased sensitivity, increased hostility and reduced involvement
- New personality characteristics: withdrawing/avoiding/isolation (from social situations, work or family), indecision, cynicism, raging attacks, self-criticism, irritability
- Somatic symptoms: digestive problems, changes in appetite and weight, diarrhea, constipation, headache, tooth pain, nausea and insomnia
- Use of alcohol and/or other drugs.

1. W**hat types of treatment are suitable for men experiencing paternal depression? (multiple answers possible)**

- Antidepressant medication
- See people
- Family support
- Talking and listening, with anyone
- Join a support group
- Support of a midwife/nurse
- Seeking help from a GP
- Seeking help from an obstetrician
- Seeking help from a psychologist
- Seeking help from a coach
- Household support
- Childcare support
- Exercise/sports
- Rest/relaxation/devote time to themselves
- Improved diet
- Sleep

1. **If you (or your husband) had postnatal depression, who would be your first choice to go to? (who would you recommend they go to first? Pick one)**

- Family or and friends
- Support group (online and offline)
- Coach
- Midwife
- Nurse
- GP
- Obstetrician
- Psychologist
- Psychiatrist
- Would not seek help
- Do not know
- Other

**PART 3: ATTITUDES AND BELIEFS**

In this section, we are looking at the attitudes and believes of the population about paternal depression.

| Q:6 Indicate to what extent you agree with the following statements.  Strongly disagree:1  Strongly agree: 6  Don’t know: missing | Strongly disagree | Disagree | Slightly disagree | Slightly agree | Agree | Strongly agree | Don’t know |
| --- | --- | --- | --- | --- | --- | --- | --- |
| 1. It is normal for men to feel depressed during his partner’s pregnancy. | 1 | 2 | 3 | 4 | 5 | 6 |  |
| 1. Paternal depression is a normal part of becoming a parent. |  |  |  |  |  |  |  |
| 1. Knowing how to look after a baby comes naturally to men. |  |  |  |  |  |  |  |
| 1. Men get perinatal depression because they cannot cope with parenthood. |  |  |  |  |  |  |  |
| 1. Men get perinatal depression because they have unrealistic expectations. |  |  |  |  |  |  |  |
| 1. Paternal depression did not exist in previous generations |  |  |  |  |  |  |  |
| 1. Paternal depression is not serious. |  |  |  |  |  |  |  |
| 1. Paternal depression requires special treatment. |  |  |  |  |  |  |  |
| 1. Paternal depression will go away on its own as the baby gets older. |  |  |  |  |  |  |  |
| 1. It is only paternal depression when you want to harm or kill the child. |  |  |  |  |  |  |  |
| 1. It is only paternal depression when you’re thinking about suicide. |  |  |  |  |  |  |  |
| 1. Only career men get perinatal depression. |  |  |  |  |  |  |  |
| 1. Men whose partners are depressed are also at risk for depression. |  |  |  |  |  |  |  |
| 1. All men should be checked for depression during pregnancy. |  |  |  |  |  |  |  |
| 1. All men should be checked for depression after the baby is born. |  |  |  |  |  |  |  |
| 1. Men should only be checked for depression when their partner is depressed. |  |  |  |  |  |  |  |
| 1. Men who take medication for paternal depression are weak-willed. |  |  |  |  |  |  |  |
| 1. Men with postnatal depression can’t be good fathers |  |  |  |  |  |  |  |
| 1. Paternal depression is a sign of weakness. |  |  |  |  |  |  |  |
| 1. Men choose to get paternal depression. |  |  |  |  |  |  |  |
| 1. Men with paternal depression just want their partner's attention. |  |  |  |  |  |  |  |

**PART 4**: **DEMOGRAPHIC DATA**

1. **What is your age?**

- 18-25 year
- 26-35 year
- 36-45 year
- 46-55 year
- 56-65 year
- 66-75 year
- 75 year of older

1. **What is your gender**

- Male
- Female

1. **What is your marital status?**

- Married
- Cohabiting
- Single or long distance relationship

1. **Do you have children?**

- Yes

How many?

- No

1. **What is your highest education for which you have obtained a diploma?**

- No education
- Primary level
- Secondary level
- Tertiary education (not university)
- Tertiary education (university)
- Other

1. **Do you have your own income?**

- Employed
- Self-Employed
- Welfare of Replacement income
- No income
- Student
- Retired

**Postal code (free space)**

1. **Which language is your mother tongue (It is about which language you speak most. This can be more than one. You have to speak all the indicated languages fluently.)?**

- Dutch
- French
- English
- Arabic
- Other

1. **Did you have any training in the field of mental health?**

- Yes
- No

1. **Have you ever had mental health problems?**

- Yes

Depression related to the perinatal period (during the first 12 months after the birth of your child)

Depression not related to the perinatal period (during the first 12 months after the birth of your child)

Other

- No

1. **Did you ever hear of paternal depression before completing this questionnaire?**

- Yes
- Studies/work
- Media
- Friends or family
- Other
- No

**Depression in Dads Questionnaire (DDads)**

DDads (Depression in Dads) Questionnaire aims to identify the awareness, knowledge and attitudes of the general population toward paternal perinatal depression. DDads questionnaire comprises three components with six questions. Component (1) “Awareness”: two open-ended questions with a maximum of four answer options for each question, Component (2) “Knowledge”: two multiple-choice questions (5 items; 16 items) with multiple answer and one multiple-choice question (12 items) with one answer option only, Component (3) “Attitudes and beliefs”: one question to rate responder’s agreement using a Likert scale on 21 attitudes and beliefs statements about paternal depression.
